# New Implications of Patients’ Sex in Today’s Lung Cancer Management

**DOI:** 10.3390/cancers14143399

**Published:** 2022-07-13

**Authors:** Jo Raskin, Annemiek Snoeckx, Annelies Janssens, Charlotte De Bondt, Reinier Wener, Mick van de Wiel, Jan P. van Meerbeeck, Evelien Smits

**Affiliations:** 1 Department of Thoracic Oncology, MOCA, Antwerp University Hospital, University of Antwerp, Drie Eikenstraat 655, 2650 Edegem, Belgium; jo.raskin@uza.be (J.R.); charlotte.debondt@uza.be (C.D.B.); reinier.wener@uza.be (R.W.); mick.vandewiel@uza.be (M.v.d.W.); jan.vanmeerbeeck@uza.be (J.P.v.M.); 2 Department of Radiology, Antwerp University Hospital, University of Antwerp, Drie Eikenstraat 655, 2650 Edegem, Belgium; annemiek.snoeckx@uza.be; 3 Center for Oncological Research (CORE), Integrated Personalized and Precision Oncology Network (IPPON), University of Antwerp, Universiteitsplein 1, 2610 Antwerpen, Belgium; evelien.smits@uantwerpen.be

**Keywords:** sex, gender, lung cancer, epidemiology, screening, immunotherapy

## Abstract

**Simple Summary:**

We aim to raise awareness that sex is an important factor to take into account in modern-day thoracic oncology practice. Summarized, women should be specifically targeted in smoking cessation campaigns and sex-specific barriers should be addressed. Women present more often with adenocarcinoma histology and EGFR/ALK alterations, as lung cancer in never-smokers is more common in women compared to men. Lung cancer in female patients may show a poorer response to immune checkpoint inhibition; therefore, the addition of chemotherapy should be considered. On the other hand, women experience more benefits from targeted therapy against EGFR. In general, prognosis for women is better compared to that in men. Lung cancer screening trials report that women derive more benefit from screening, although they have not been designed for women. Future trial designs should take this into account and encourage participation of women.

**Abstract:**

This paper describes where and how sex matters in today’s management of lung cancer. We consecutively describe the differences between males and females in lung cancer demographics; sex-based differences in the immune system (including the poorer outcomes in women who are treated with immunotherapy but no chemotherapy); the presence of oncogenic drivers and the response to targeted therapies according to sex; the greater benefit women derive from lung cancer screening and why they get screened less; and finally, the barriers to smoking cessation that women experience. We conclude that sex is an important but often overlooked factor in modern-day thoracic oncology practice.

## 1. Introduction

Lung cancer is highly prevalent worldwide. It is the second most common cancer in men and the third most common cancer in women. It is also the most lethal cancer in both men and women. In women, its mortality supersedes that of breast, uterine, and ovarian cancer combined [1,2].

The terminology is often misused when it comes to sex and gender. Sex is defined by biological traits such as chromosomes, hormones, and primary and secondary sexual characteristics. Gender indicates how somebody identifies and may differ from their biological sex. This article defines men and women on the basis of biological sex and does not take into account gender.

Today, cancer stage (TNM), age, performance status, and histology have been expanded with programmed-death ligand-1 (PD-L1) expression and the absence or presence of a targetable oncogenic driver as factors that may impact treatment decisions in non-small cell lung cancer (NSCLC). Small cell lung cancer (SCLC) is another type of lung cancer with its own treatment strategies. Sex is usually neglected during treatment decisions and findings in males have often been generalized to females with the assumption that males and females are biologically and physiologically identical [3].

The European Society for Medical Oncology (ESMO) recently published a consensus report that highlights the dominance of men in most cancer types [4]. This report underlines the importance of awareness regarding sex in daily practice in order to optimize treatment for all patients. It has only been since 2014 that the National Institutes of Health (NIH) invited researchers to consider sex a biological variable in research [3,5]. Attention given to the aspect of sex in health research has been one of the main initiatives of EU policy in the GenderBasic project [6].

This article discusses where and how sex matters in today’s lung cancer practice. We describe the differences between lung cancer in male and female patients, the different impacts of smoking and radiation, how sex plays a role in the recently approved first-line treatments of lung cancer, and the potential disparities in lung cancer screening and smoking cessation.

## 2. Sex and Lung Cancer Epidemiology

Lung cancer is the third most common cancer in women, after breast and colon cancer, but by far the most lethal [2]. Women have clearly caught up to men in the past decades, both in smoking and the resulting increase in lung cancer diagnoses. Anti-smoking campaigns have caused the incidence of lung cancer and the resulting mortality to decrease in men in most developed countries. For women, on the contrary, the incidence of lung cancer is rising, as is mortality [7,8,9].

Currently, adenocarcinoma is the most prevalent histologic subtype of NSCLC, both in men and women. In women, up to 80% of lung cancers are adenocarcinomas, as opposed to only 60% in men, which is attributable to differences in smoking habits. Cancer in never-smokers is also more common in females. The likelihood of a lepidic-predominant adenocarcinoma (the former bronchoalveolar carcinoma; cancer cells spreading along the alveolar wall) is increased 2–4-fold in women [9,10]. There does not seem to be a distinction between the sexes when it comes to small cell lung cancer [11].

The 5-year survival rate across all stages of lung cancer was 19.7% in men and 27.8% in women (2014–2018), regardless of their stage, age, or histologic subtype [1,8,10]. This difference cannot be explained by the fact that women have a longer life expectancy in general. This might be partly explained by the earlier diagnosis of lung cancer in women, differences in histology, fewer comorbidities, better response to chemotherapy (but possibly at the cost of higher toxicity), and better prognosis in non-smokers’ lung cancer [12,13,14].

### Smoking in Females

Female smokers are more likely to develop lung cancer compared to their male counterparts when they smoke the same number of cigarettes [11,15]. Several explanations have been given for this difference; different lifestyles and smoking behaviors of men and women, the smaller lung volume and different airway behavior in females, and genetic polymorphisms in detoxifying enzymes may all contribute to greater carcinogen exposure in women [16]. Controversy remains over whether female sex hormones play a role in the development of lung cancer regardless of smoking status [17]. Estrogen receptors are present in both normal and neoplastic lung tissues and could accelerate the metabolism of smoke-related carcinogens in a dose-dependent way, as suggested by higher levels of polycyclic aromatic hydrocarbons–DNA adducts in female smokers compared to males [18,19]. Several reviews have addressed these sex-based differences [10,20,21,22,23].

## 3. Sex and Cancer Immunity

A recent meta-analysis observed that, in general, female cancer patients had a lesser benefit of checkpoint inhibition across cancer types. This included females with NSCLC (pooled OS hazard ratio of 0.89 (0.71–1.11)) as opposed to male patients (pooled hazard ratio of 0.72 (0.61–0.86)) [24]. Sex differences in the immune response seemed to be responsible for this difference.

### 3.1. The Immune System in Female Patients

The response to immunotherapy is influenced by many factors, some yet to be discovered, but the intrinsic characteristics of the tumor and of the tumor environment are key in provoking an immune response [25]. Sex differences are also applicable in the immune system. In general, women have stronger innate and adaptive immune responses than men, which is illustrated by the higher incidence of autoimmune diseases in women [26]. The immune system differs between males and females due to genetic differences and sex-specific levels of hormones (estradiol, progesterone, and androgens).

The X chromosome contains several immune-related genes with different inheritance patterns for women and men [27]. Nearly all immune cells express receptors for sex hormones that may influence the expression of several immune-related genes via responsive elements in promotor sites [28]. Progesterone, although dependent on its concentration, has anti-inflammatory effects. Androgens suppress immune cells. Estradiol improves cell-mediated and humoral immune response. Low estrogen levels tilt the T helper (Th) response towards Th1 differentiation, enhancing cellular immunity. High estrogen levels shift the equilibrium towards the Th2 phenotype [25].

Currently approved immunotherapies in NSCLC are monoclonal antibodies against the PD-1 (nivolumab, pembrolizumab, and cemiplimab), the PD-L1 (atezolizumab and durvalumab), or CTLA-4 proteins (ipilimumab). PD-1 is a cell surface receptor present on pro B and T lymphocytes and plays a role in downregulating the immune response and self-tolerance when binding to its ligands PD-L1 and PD-L2. This aids in preventing autoimmunity, but cancer cells may upregulate PD-L1 to escape immune-mediated elimination. PD-1 expression is influenced by estrogen and prolactin, and therefore, is sex-dependent [29]. PD-L1 expression on tumor cells, on the contrary, should be less sensitive to the hormonal surroundings of the host. CTLA-4 is another protein receptor that is upregulated in activated T cells and responsible for suppressing the activity of other T cells [30].

### 3.2. Immune Checkpoint Inhibition in Female Patients

The first-line treatment of stage IV non-small cell lung cancer has recently been altered by several immunotherapy trials (Table 1 and Table 2). In these trials, in which the experimental arm did not contain chemotherapy (currently approved for high (≥50%) PD-L1 expression and, in some countries, also for ≥1% PD-L1 expression), we noticed that the hazard ratio for overall survival was consistently lower (and even non-significant) in female patients compared to male patients (Table 1).

The landmark KEYNOTE-024 trial compared pembrolizumab to platinum-based chemotherapy in treatment-naive patients. With a hazard ratio (HR) of 0.95 (95% CI 0.56–1.62) in females and 0.54 (95% CI 0.36–0.79) in males, this trial did not show a significant survival benefit in female patients [31]. The similar EMPOWER-Lung 1 trial compared cemiplimab to chemotherapy with identical findings: in females, the hazard ratio was insignificant at 1.11 (95% CI 0.42–2.59) (males: 0.50; 95% CI 0.36–0.69) [32]. The KEYNOTE-042 trial allowed for the lower PD-L1 expression of ≥1%. Again, pembrolizumab was superior to chemotherapy in men, with an OS hazard ratio of 0.68 (95% CI 0.53–0.88), but not in women (HR 0.78; 95% CI 0.53–1.15; results for the PD-L ≥ 50% subgroup as well as for the entire population) [33]. Finally, the IMPOWER-110 trial also included PD-L1-positive patients (≥1%), comparing atezolizumab to chemotherapy. In the TC3/IC3 subgroup, the hazard ratio for women was 0.69 (95% CI 0.34–1.39), in contrast to the male ratio of 0.57 (95% CI 0.35–0.93) [34]. Median OS values according to sex were only reported in (the appendix of) this last trial: 23.1 months for males (gaining a median of 10.0 months) and 17.8 months for females (gaining a median of 3.7 months). These findings were observed by a recent meta-analysis [35].

In addition to this, the CHECKMATE-227 trial investigated an immunotherapy combination of nivolumab plus ipilimumab in PD-L1-positive patients (≥1%), with the chemo-free arm outperforming chemotherapy in males (HR 0.75; 95% CI 0.61–0.93), but not in females (HR 0.91; 95% CI 0.69–1.21) [36]. It appears that women, unlike men, do not benefit (as much) from immunotherapy, whether in monotherapy or combined. In addition to fitness for chemotherapy and the need for a rapid response, biological sex might also be a criterion to consider when selecting first-line therapy in PD-L1-high NSCLC.

In patients with moderate (1–49%) and low (<1%) PD-L1 expression, immunotherapy combined with chemotherapy is the current standard of care. Here, the results of female and male subgroups are different. The two pembrolizumab trials showed an OS benefit in both males and females—females clearly more with non-squamous and males slightly more with squamous histology (Table 2) [37,38]. A second meta-analysis of immunotherapy, alone or in combination with chemotherapy, confirmed that women derived a higher benefit compared to men from the combination of pembrolizumab/chemotherapy versus chemotherapy or any other treatment option (all P_interaction_ < 0.02) [39].

In the IMPOWER-130 and -131 trials, females had a greater benefit in both histologies [40,41]. In contrast, in the IMPOWER-150 trial, which added bevacizumab to the chemotherapy backbone, no real benefit was found in female patients [42]. Finally, in the CHECKMATE-9LA trial, no clear difference in HR was observed [43]. The proportion of female patients in all these trials was very heterogeneous: from 12% in the EMPOWER-Lung 1 trial to >40% in the IMPOWER-130 and KEYNOTE-024 trials, and statistical considerations of subgroup analysis may apply.

The fact that women are at an advantage with the addition of chemotherapy, as opposed to treatment with single-agent immunotherapy, could be the result of a greater mutational burden and tumor antigenicity in men. The recent EMPOWER-Lung 1 trial demonstrated that even within the high PD-L1 category, there were differences in response according to the PD-L1 level [32], but there was no clear evidence that the average PD-L1 expression was lower in females [44].

Considering the adverse events of immunotherapy, the female sex has been reported to be associated with greater toxicity of checkpoint inhibitors when inhibiting both CTLA-4 and PD-1/PD-L1 [45,46,47].

**Table 1:** List of phase 3 trials of approved immunotherapy schemes not containing chemotherapy in first-line treatment of non-small cell lung cancer.

			Women	Men
	Histology	PD-L1	HR	HR
KEYNOTE-024 [31]	All	≥50%	0.95 (0.56–1.62)	0.54 (0.36–0.79)
KEYNOTE-042 [33]	All	≥1% *	0.89 (0.68–1.17)	0.80 (0.68–0.94)
IMPOWER-110 [34]	All	≥1%	0.69 (0.34–1.39)	0.57 (0.35–0.93)
EMPOWER-Lung 1 [32]	All	≥50%	1.11 (0.42–2.59)	0.50 (0.36–0.69)
CHECKMATE 227 [36]	All	≥1%	0.91 (0.69–1.21)	0.75 (0.61–0.93)

* This trial included patients with PD-L1 expressions >1%; subgroup with PD-L1 ≥ 50% reported in the plain text. HR = hazard ratio.

**Table 2:** List of phase 3 trials of approved immunotherapy schemes containing chemotherapy in first-line treatment of non-small cell lung cancer.

			Women	Men
	Histology	PD-L1	HR	HR
KEYNOTE-189 [37]	NSQ	All	0.29 (0.19–0.44)	0.66 (050–0.87)
KEYNOTE-407 [38]	SQ	All	0.49 (0.30–0.81)	0.42 (0.22–0.81)
IMPOWER-130 [40]	NSQ	All	0.66 (0.46–0.93)	0.87 (0.66–1.15)
IMPOWER-131 [41]	SQ	All	0.68 (0.44–1.04)	0.91 (0.75–1.12)
IMPOWER-150 [42]	NSQ	All	0.92 (0.70–1.22)	0.72 (0.58–0.90
CHECKMATE 9LA [43]	All	All	0.68 (0.47–1.00)	0.66 (0.53–0.82)

HR = hazard ratio; NSQ = non-squamous; SQ = squamous.

Immunotherapy in localized and locoregional stages falls beyond the scope of this paper, but the poorer efficacy of immunotherapy in women might be a factor to consider when choosing between neoadjuvant immunotherapy or neoadjuvant immunochemotherapy.

## 4. EGFR and ALK Inhibition in Females

Cancer in never-smokers is more common in females; in general, adenocarcinoma histology (NSCLC) is detected [11]. Often, an oncogenic driver is identified in these patients. Mutations in the epithelial growth factor receptor (EGFR) are the most common driver in never-smokers and are more often found in women (odds ratio of 2.7; 95% CI 2.5–2.9) [3], in addition to Asians and Caucasians and those with adenocarcinoma histology [48]. For this reason, women are more often treated with targeted therapies and seem to benefit more compared to men in EGFR-mutated lung cancer. This is not the case in lung cancer with an anaplastic lymphoma kinase (ALK) fusion, where survival data are comparable between men and women [49].

## 5. Sex and Lung Cancer Screening

In the past decade, randomized controlled trials have shown that lung cancer screening (LCS) with low-dose computed tomography (LDCT) could reduce lung-cancer-specific mortality and save lives (Table 3). To maximize effectiveness and minimize potential harms, LCS targets individuals at the highest risk of developing lung cancer—those who are generally] older and (formerly) heavy smokers. Women are underrepresented in LCS trials, with participation rates ranging from 0% to 44.8% [49,50]. The Italian DANTE-trial only included male participants [51]. At the start of the Dutch–Belgian NELSON trial, only male participants were included [50]. Further on in the recruitment, the trial was also opened to women, with a final 16.4% of female participants. The participation of women in different LCS trials is reported in Table 3.

### 5.1. Benefits from Lung Cancer Screening (LCS) in Female Patients

Although LCS trials were not specifically designed for women, LCS trials have revealed differences in lung cancer-specific mortality, LCS being far more beneficial in women than in men. Three randomized lung cancer screening trials have stratified the outcome data by gender: NLST, LUSI, and NELSON.

In the North American National Lung Screening Trial (NLST), the rate ratio for mortality from lung cancer among female participants in the LDCT group, as compared to those in the chest-radiography group, was 0.80 (95% CI, 0.66 to 0.96) for a follow-up period of 12.3 years [58]. The German Lung Cancer Screening Intervention Trial (LUSI) showed a significant benefit with respect to lung cancer mortality in the small subgroup of women who were invited to undergo screening (HR 0.31, 95% CI, 0.10 to 0.96) [57]. The final publication of the Dutch–Belgian Lung Cancer Screening Trial (NELSON) mainly focused on the results of men due to the low number of women involved in the trial. Although not statistically significant, the data on the small subset of women showed more favorable effects of LCS for women than for men, with a rate ratio for death from lung cancer of 0.67 (95% CI, 0.38 to 1.14) at 10 years of follow-up. The magnitude of lung cancer-specific mortality reduction in women was even greater at 7, 8, and 9 years from baseline [59].

A meta-analysis by Hoffman et al. [60] revealed that women benefitted substantially more from screening, with a 31% relative risk reduction in lung cancer mortality compared to 14% in men. Risk reductions were statistically not significant (*p* = 0.11). As previously mentioned, women were underrepresented in these LCS trials, so analyses for women were likely underpowered.

The inclusion criteria for LCS take into account the number of pack-years reflecting the severity of smoking history. In general, women accumulate fewer pack-years than men, resulting in differences in eligibility for LCS. Modeling studies have shown that expanding eligibility to include ever-smokers with less than 30 pack-years of exposure (20–29 pack-years) would not only increase the proportion of lung cancer deaths prevented by screening but would also reduce disparities in eligibility by sex [61].

### 5.2. Harms of Lung Cancer Screening in Female Patients

Using low-dose CT, LCS is performed at radiation doses much lower than doses used in clinical practice for diagnostic chest CT imaging. Despite the lower radiation dose, radiation remains one of the harms associated with LCS. Our knowledge from long-term studies on the impact of these levels of radiation exposure on cancer risk is very limited.

There is an interaction between radiation and smoking, with cancer risk from radiation generally being higher in the target population of smokers and former smokers. In addition to smoking and age, sex plays a role in the estimated risks of lung cancer associated with radiation. Excess relative risk differs between men and women and is higher in women than in men. Brenner et al. calculated the risks for yearly LDCT lung cancer screening: yearly screening would result in a 5% increase in the risk of lung cancer in women. In men, this increased risk would only be 1.5% [62]. The precise mechanisms underlying the sex differences in radiation-induced cancers remain unknown. The roles of hormonal regulation, genetic risks, and X-linked factors still need to be determined [63,64].

Rampinelli et al. retrospectively investigated the cumulative radiation exposure and lifetime attributable risk of cancer incidence associated with LDCT from a 10-year lung cancer screening program. They showed that the lifetime attributable risk of lung cancer was estimated to be about four times greater for women aged 50–54 years than for men aged 65 and older. The risk for other major cancers is up to three times greater. Both the increased radiosensitivity of women and the risk of breast cancer associated with chest imaging are postulated to be the cause of this difference [65].

Overdiagnosis is another harm in LCS. Cancer overdiagnosis is the detection of asymptomatic cancers that would never have caused medical problems or harm during the patient’s lifespan because of death from other causes [66]. In the era of LCS, it is defined as screen-detected cancer that would not have become symptomatic during a person’s lifetime. Blom et al. estimated overdiagnosis in lung cancer screening using the cumulative excess-incidence approach. With this approach, the difference in cumulative incidence between a screened group and a matched control group is attributed to overdiagnosis. Overall, the percentage of overdiagnosis of screen-detected cancers was higher in women (ranging from 5.7% in the 1990 cohort to 11.2% in the 1950 cohort) than in men (ranging from 61% in the 1990 cohort to 9.8% in the 1950 cohort) in all cohorts except the 1990 cohort. An explanation of this overdiagnosis may be related to the predominant slower-growing adenocarcinoma histology in women. The longer the preclinical duration of the disease, the higher the likelihood of overdiagnosis [67].

### 5.3. Eligibility and Uptake of Lung Cancer Screening in Female Patients

Lung cancer screening targets high-risk participants, with smoking history being the most important risk factor. Smoking habits vary between sexes, with sex differences also varying between countries [68]. Differences in current smoking habits will impact trends in lung cancer incidence in the upcoming decades. Currently, most screening programs are ‘one-size-fits-all’, with no different eligibility criteria for women and men. The risk of disease depends, however, on many individual factors, including sex and age.

A comparative simulation modeling study investigating seven selected risk factor-based screening scenarios showed a lower percentage of eligibility for women in all scenarios. In contrast, for all except one scenario, the percentage of mortality reduction was higher in women. The number of people needed to (ever) screen to prevent one lung cancer death (NNS) was lower for women compared to men for all scenarios. Due to radiation sensitivity in women (as discussed previously), the number of radiation-related lung cancer deaths was higher in women than in men for all seven scenarios [61]. Lung cancer tends to be diagnosed in women at a younger age, an aspect that is not taken into account in the selection criteria for LCS [69].

Unlike other types of cancer screening targeting healthy populations based on age and/or sex, lung cancer screening targets a population with poor health habits, namely tobacco smoking. Targeting this group remains one of the biggest challenges in lung cancer screening [70]. Recent data for 10 states in the United States show an uptake of 14.4% of the population eligible for lung cancer screening. Uptake by high-risk women was slightly less (13.8%) compared to high-risk men (14.8%) [71]. A recent systematic review and meta-analysis by Lopez-Olivo et al. [72] examined adherence and diagnostic testing rates after LCS in the US. The data on sex as a patient characteristic associated with adherence rates were limited to 4 out of 15 studies. Sex was not a patient characteristic that was statistically significantly associated with LCS adherence (OR 1.0; 95% CI 0.8–1.3). The absence of differences in nonadherence rates between males and females was also demonstrated by Lam et al. (RR 0.99; 95% CI 0.85–1.15; *p* = 0.85) [73]. Raju et al. investigated the characteristics and barriers of nonparticipants to LCS. Their retrospective study demonstrated that being female was associated with not participating. Furthermore, women were more likely to identify the distance to the main screening center as a significant barrier to LCS participation (OR 22.5%; 95% CI 2.86–176.67; *p* = 0.003). Potential reasons that were not studied include the differences in awareness of lung cancer risk in women or the role of increased screening burden for other cancers; breast, cervical, and colon cancer screening are also recommended for women in this age group [74]. Barton et al. reported in 2015, two years after the recommendation for LCS through the US Preventive Services Task Force (USPSTF), that women perceived the benefits of LCS as much lower (42%) compared to screening for other cancers, including breast cancer with mammography (93%), colon cancer with colonoscopy (99%) and screening for cervical cancer with Pap smear (96%) [75,76].

For decades, women have been familiar with image-based screening through breast cancer screening programs. Today, screening with mammography is widely accepted by women. Although one might assume that this experience would positively impact the participation of women in LCS, unfortunately, this is not the case. A study by Lopez et al. showed that 7.1% of women undergoing mammography within the last 2 years were eligible for lung cancer screening. Among these LCS-eligible women, only 8.0% reported receiving LCS. As far as the limited data suggest, the uptake of lung cancer screening in this group of women who undergo breast cancer screening is not higher than that in the general population. Mammography programs do represent an opportunity for health care providers to identify and motivate women for LDCT LCS. Targeted education efforts could focus on this group since they seem to be convincible of the benefits of screening [77].

### 5.4. Future Challenges and Opportunities for Female Patients

Although current LCS trials suggest a larger benefit of LCS for women than men, further research is needed to evaluate and confirm the observed sex differences, in particular, the marked lung cancer survival benefits for women. To optimize the effectiveness of LCS programs, the barriers to the participation of women must be identified. Recruitment resources should focus on all eligible individuals, but future recruitment strategies may be specifically focused on the participation of women. Smoking trends are different for men and women, and it is expected that in the future, more lung cancers will occur and be detected in women. This addresses the need for a women-tailored approach to LCS. The incorporation of risk prediction models to address eligibility for screening might overcome the challenges of eligibility through the incorporation of specific sex-related factors in these models.

The 2020 Official American Thoracic Society Statement on Addressing Disparities in Lung Cancer Screening Eligibility and Healthcare Access also addresses the topic of sex and eligibility for LCS. It states that addressing sex-based differences in smoking behaviors and lung cancer risk is important for the eligibility of future lung cancer screening. It is important to improve the overall health of the U.S. population and reduce healthcare costs [78]. The recently published new and revised USPSTF guidelines [79] specifically highlight the need for research regarding the benefits and harms of lung cancer screening in more diverse community settings, including settings that screen greater numbers of women.

## 6. Smoking Cessation in Female Patients

The key to the prevention of lung cancer is smoking cessation. Therefore, participants in LCS programs should be encouraged to quit smoking and offered help to do so. Even after lung cancer diagnosis, smoking cessation is beneficial. In early-stage patients who were treated with curative surgery or radiotherapy, smoking cessation may prevent the development of a second primary tumor. Active smokers also have more complications with lung cancer surgery and pulmonary infections [80]. In addition, nicotine has been reported to increase resistance to chemotherapy, so even for stage IV disease, smoking cessation may be advantageous [81].

Female patients who quit smoking showed 2.5 times greater improvement in FEV_1_ compared to males, whereas annual FEV_1_ decline is proportionally greater in women who persisted in their smoking habit [82]. Abstaining from smoking is therefore even more important in female patients.

Browning et al. observed that health care providers did not differ in their assistance in smoking cessation in males and females [83], but several studies report that women may find it harder to quit than men, feel less motivation to quit, have more doubts about their ability to quit successfully, and experience more stress during the quitting process. Women also reported more symptoms of nicotine withdrawal than men [84].

Men appeared to have a greater physical dependence on smoking, whereas women have a greater behavioral dependence, which may cause nicotine replacement therapy to be less effective in women [85]. Specific barriers to smoking cessation may exist in women, in particular, the fear of weight gain, which may be addressed with psychotherapy [86]. The menstrual cycle may also play a role in smoking cessation; quitting during the follicular phase has been reported to be less effective compared to the luteal phase [87].

## 7. Conclusions

Differences between men and women in today’s lung cancer management are often neglected, despite their importance. Lung cancer incidence and mortality are rising in women; women should therefore be specifically targeted in smoking cessation campaigns, and sex-specific barriers should be addressed. Women present more often with adenocarcinoma histology and EGFR/ALK alterations, as lung cancer in never-smokers is more common in females. Women may be more susceptible to lung cancer, but the role of female sex hormones is still controversial. Lung cancer in female patients may show a poorer response to immune checkpoint inhibition; therefore, the addition of chemotherapy should be considered. On the contrary, women experience more benefits from targeted therapy against EGFR. In general, prognosis for women is better compared to that in men. Finally, lung cancer screening trials report that women derive more benefit from screening, although they have not been designed for women. Future trials should focus on women and encourage participation.

## Figures and Tables

**Table 3:** Percentage of women in lung cancer screening trials.

Lung Cancer Screening Trial	% Females
Danish Lung Cancer Screening Trial (DLCST) [52]	44.8
Lung Screening Study (LSS) [53]	41.4
National Lung Screening Trial (NLST) [54]	41.0
Multicentric Italian Lung Detection (MILD) [55]	35.5
Italian Lung Study (ITALUNG) [56]	35.3
German Lung Cancer Screening Intervention (LUSI) [57]	35.3
Nederlands Leuvens Longkanker Screeningsonderzoek (NELSON) [50]	16.4
Detection and Screening of Early Lung Cancer with Novel Imaging Technology and Molecular Assays (DANTE) [51]	0
